# Time loss due to dental problems and treatment in the Canadian population: analysis of a nationwide cross-sectional survey

**DOI:** 10.1186/1472-6831-13-17

**Published:** 2013-04-15

**Authors:** Alyssa Hayes, Amir Azarpazhooh, Laura Dempster, Vahid Ravaghi, Carlos Quiñonez

**Affiliations:** 1 Discipline of Dental Public Health, Faculty of Dentistry, University of Toronto, Toronto, ON, Canada; 2 Oral Health & Society Research Unit, Faculty of Dentistry, McGill University, Montreal, QC, Canada

**Keywords:** Cost of illness, Economics/Dental, Oral health, Socio-demographic/Economic

## Abstract

**Background:**

The purpose of this study was to quantify time loss due to dental problems and treatment in the Canadian population, to identify factors associated with this time loss, and to provide information regarding the economic impacts of these issues.

**Methods:**

Data from the 2007/09 Canadian Health Measures Survey were used. Descriptive analysis determined the proportion of those surveyed who reported time loss and the mean hours lost. Linear and logistic regressions were employed to determine what factors predicted hours lost and reporting time loss respectively. Productivity losses were estimated using the lost wages approach.

**Results:**

Over 40 million hours per year were lost due to dental problems and treatment, with a mean of 3.5 hours being lost per person. Time loss was more likely among privately insured and higher income earners. The amount of time loss was greater for higher income earners, and those who reported experiencing oral pain. Experiencing oral pain was the strongest predictor of reporting time loss and the amount of time lost.

**Conclusions:**

This study has shown that, potentially, over 40 million hours are lost annually due to dental problems and treatment in Canada, with subsequent potential productivity losses of over $1 billion dollars. These losses are comparable to those experienced for other illnesses (e.g., musculoskeletal sprains). Further investigation into the underlying reasons for time loss, and which aspects of daily living are impacted by this time loss, are necessary for a fuller understanding of the policy implications associated with the economic impacts of dental problems and treatment in Canadian society.

## Background

It is estimated that oral diseases affected over 95 percent of Canadian adults in 2009 [1]. Almost 20 percent of Canadian adults had untreated coronal decay, while approximately 20 percent had moderate to severe periodontal disease [1]. That same year, Canadians spent approximately $12.8 billion for dental care [1]. Repre- senting about ten percent of overall health care spending in Canada, the direct costs of dentistry are said to rank second only to those for cardiovascular diseases [1]. Within health economics, direct costs represent the resources consumed to treat a condition and can include health care sector resources, out-of-pocket expenses and sometimes funds from statutory or voluntary bodies [2]. In contrast, indirect costs represent time (often work time) consumed for treatment and is synonymous with ‘productivity losses’ [2].

The overall costs of oral disease encompass both direct and indirect costs. The direct costs are attributed to care provided by dental professionals, while the indirect costs are attributed to time loss from work, school or normal activities due to dental problems and treatment. Current estimates on the direct costs of dental care are significant, yet there has been a dearth of estimates regarding the indirect costs. The recently completed Canadian Health Measures Survey (CHMS) provides an opportunity to estimate the indirect costs of dental problems and treatment in Canadian society. Previous research in this area has shown that, as ‘socio-dental indicators,’ the use of time loss from work, school or normal activities allows for dental problems and treatment to be understood in terms of impaired role functioning leading ultimately to potential productivity losses. Time loss is also easily operationalized, and when combined with wage information, can help estimate the economic impacts that oral diseases have on society. Quantifying time loss and the associated potential productivity losses thus allows for policy discussions to focus on the total burden of illness among different diseases and not merely the clinical aspects of any given disease [3].

Work in this area has been completed in other Organization for Economic Cooperation and Development (OECD) countries (i.e., the United States and Australia). For example, the U.S. Surgeon General (2000), using a question from the 1996 National Health Interview Survey which asked about time loss within a previous 2-week period, reported an estimated 3.7 restricted activity days per 100 persons but did not quantify these losses monetarily [4]. In comparison, several estimates are available for Australia. The Australian Research Centre for Population Oral Health (ARCPOH) questioned individuals about time loss within a previous 12-month period, and combined this with wage information to estimate potential productivity losses. For example, losses due to oral pain and discomfort were estimated at $836.5 million CDN [5].

In short, quantifying the impacts of dental problems and treatment monetarily allows governments to more clearly understand the overall burden of illness in the population and allows oral health to be included and compared to other diseases in the broader health policy debate. Thus, the purpose of this study was to quantify time loss due to dental problems and treatment in the Canadian population, to identify factors associated with this time loss, and to provide information regarding the economic impacts of these issues.

## Methods

### Study design and sample

This study was a secondary analysis of the 2007/09 Canadian Health Measures Survey (CHMS). Statistics Canada designed the CHMS to be a voluntary, nationally representative cross-sectional survey aiming to collect direct health measures from Canadians aged 6 to 79 years who resided in private households [6,7]. Those living in institutions, on crown land or Indian reserves, in remote regions and full-time members of the Canadian Forces were excluded [7]. Exclusions were largely due to logistic concerns regarding travel to mobile examination clinics [8]. In terms of Aboriginal populations living on crown land or Indian reserves, separate targeted surveys were completed in order to comply with data ownership agreements. Ethical review and consultation regarding the survey’s ethical, social and legal issues was provided by Health Canada’s Research Ethics Board, the Office of the Privacy Commissioner of Canada, and the Data Access and Control Services Division at Statistics Canada [9]. Four consent forms were used for this survey: a consent form for respondents aged 20 to 79 years, for respondents aged 14 to 19 years and for parents/guardians of respondents aged 6 to 13 years, and an assent form for respondents aged 6 to 13 years [9]. Further information can be found in Day, Langlois, Tremblay and Knoppers (2007) [9].

The CHMS employed a multi-stage sampling method that incorporated both dwelling and physical data collection, and can be reviewed elsewhere [6,7]. Data were collected from 5,586 Canadians, which when weighted represented 29,157,460 Canadians out of a current population of 33,476,688, statistically representing 97 percent of Canadians aged 6 to 79 years [10]. This provided national estimates for five age groups, which were equally distributed for age and sex (6 to 11, 12 to 18, 20 to 39, 40 to 59, and 60 to 79) for a total of 10 groups [7]. National estimates for conditions with a prevalence of 10 percent or higher and a coefficient of variation (CV) of 16.5 percent were obtained, where the CV represents the estimated standard error percentage of the survey estimate [8]. To ensure data were nationally representative the CHMS required that 500 bootstrap weights be used over the 10 age-sex groups [8]. Both the sample and bootstrap weights were applied in Stata v12.0 prior to analysis [11].

Data collection comprised two stages, a household questionnaire and clinical examination and was conducted between March 2007 and March 2009. The household questionnaire consisted of 47 modules (722 questions) focusing on health status, nutrition and food, medication use, health behaviours, and socio-economic information [12]. The computer-aided questionnaire was aided by a Statistics Canada interviewer [7]. Within six weeks of the household interview, the clinical exam was conducted for those selected, and occurred in a mobile clinic where physical measures (cardiovascular fitness, oral health exam, anthropometry, muscle strength and flexibility), blood, and urine samples were gathered [7]. Oral health data were collected in both the household questionnaire and the clinical examination. Within the questionnaire there were 34 oral health related questions pertaining to oral health satisfaction, dental care habits, oral symptoms and disability days [1]. The oral health exam began with a further 18 questions regarding symptoms (bleeding, pain, xerostomia, etc.) followed by a clinical exam completed by Canadian Forces dentists calibrated to World Health Organization (WHO) standards [1]. For this study, data were accessed through Statistics Canada’s Research Data Centre (RDC) at the University of Toronto.

### Data variables and analysis

Within the household questionnaire participants were asked: “In the past 12 months, have you taken time away from work, school or your normal activities due to check-ups, treatments or because of problems with your mouth?” If participants answered “yes” they were then asked to quantify the amount of time lost (to the nearest half hour). In this study, the first dependent variable relates to time loss from work, school and normal activities in the previous 12 months and was dichotomized (if the participant did or did not report time loss). The second dependent variable dealt with the amount of time lost by those who responded yes to the previous question, and was continuous (participants reported amount of time loss to the nearest half hour). Independent variables were organized into predisposing (e.g., sex, age, career status, etc.), enabling (e.g., insurance, income adequacy), needs (e.g., self-perceived oral and general health etc.) and health services use (e.g., frequency of seeing a dental professional, time since last dental visit) factors as per the Andersen behavioural model of health services utilization [13]. Details regarding the coding of variables, or answer options of participants can be seen in Table 1.

**Table 1:** Sample characteristics, Canadian health measures survey, 2007-2009

**Variable (N)**	**%**
Age (yrs, N=29,157,460)	
6 to 11	7.5
12 to 19	11.5
20 to 39	30.7
40 to 59	33.5
60 to 79	16.8
Sex (N=29,157,460)	
Male	49.9
Female	50.1
Educational attainment (N=28,740,821)	
Greater than high school	74.4
Less than high school	25.6
Career status (N=25,001,490)	
Employed	53.0
Student	16.2
Unemployed	30.8
Employment type (N=0.8626)	
Full-time	79.5
Part-time	20.5
Household size (N=29,157,460)	
1 to 2 people	44.0
3 to 4 people	40.7
5 or more people	15.3
Occupational classification (N=20,193,946)	
Management	8.0
Business, finance and administrative	17.7
Natural and applies sciences and related occupations	9.1
Health occupations	5.9
Occupations in social science, education, government service and religion	9.3
Occupations in art, culture, recreation and sport	4.8
Sales and service occupations	24.4
Trades, transport and equipment operators and related occupations	14.1
Occupations unique to primary industry	2.4
Occupations unique to processing, manufacturing and utilities	4.4
Insurance (N=28,976,586)	
Private insurance	62.6
Public insurance	5.5
No insurance	31.9
Income adequacy (N=27,216,855)	
Highest income	47.9
Upper middle income	31.9
Middle income	14.8
Lower middle income	3.9
Lowest income	1.6
Self-reported oral health (N=29,152,410)	
Good to excellent	84.5
Poor to fair	15.5
Self-reported oral pain (N=29,149,758)	
Rarely or never	88.4
Sometimes	9.1
Often	2.5
Treatment needs (N=29,149,991)	
Yes	67.2
No	32.8
Preventive needs (N=29,149,991)	
Yes	13.7
No	86.3
Frequency of seeing dental professional (N=29,152,032)	
Emergency or never	16.5
Less than once a year	9.2
One or more times per year	74.3
Time since last dental visit (N=28,208,528)	
Less than 1 year ago	74.5
More than 1 year ago	25.5

Descriptive statistics (frequencies, means) were used to observe the sample’s characteristics and to determine the proportion who reported time loss by selected predisposing, enabling, needs and health services use factors. Univariable logistic regressions were conducted to produce unadjusted odds ratios (OR) for reporting time loss. Multivariable logistic regression was then conducted to determine which variables were dominant predictors of reporting time loss, the variables included in the model were: age, sex, career status, occupational classification, insurance, income adequacy, self-reported oral pain, self-reported general pain, treatment needs, preventive needs, frequency of seeing dental professional, and times since last dental visit. Lastly, a multivariable linear regression was employed for the continuous outcome of hours lost, the variable included were: sex, career status, self-reported oral health, self-reported general health, and time since last dental visit. It is important to note that only variables with low multicollinearity, as represented by a variance inflation factor (VIF) of less than 3 and p-values of less than 0.25 in the univariable analyses were entered into the multivariable analyses. The VIF, which is the inverse of tolerance, represents the extent to which variances are inflated or increased due to collinearity [14]. In both the logistic and linear regressions the only variable omitted due to collinearity was self-reported mental health. As the CHMS did not collect labour force data for those aged 6 to 11 years, they were combined with the 12 to 19 year old category to form the reference group for this part of the analysis.

Potential productivity losses, represented by the socio-dental indicator of time loss from work, were monetized using the human capital or lost wages method [15]. This method assumes that wages equate to the revenue a person produces and when lost represents their foregone earnings or opportunity cost [16]. The CHMS collected labour force information pertaining to occupation classification, which corresponded to data collected in Canada’s Labour Force Survey (LFS) [17]. Average hourly wages per occupation classification (accurate as of February 2012) were used to estimate individual losses (i.e., Individual losses = mean hours lost × average hourly wages). Societal losses were estimated using individual losses attributed across the total number of employees per occupation, which were also accurate as of February 2012 (i.e., Societal losses = individual losses × number of employees).

## Results

The overall participation rate for the CHMS was 51.7 percent, meaning that of the 8,772 households selected, 69.6 percent agreed to participate, and of these, 88.3 percent completed the household questionnaire, and 84.9 percent visited the mobile examination centre [18]. Table 1 demonstrates that of those surveyed, there was an equal proportion of males (49.9%) and females (50.1%), over 60 percent were between the ages of 20 and 59 years, and 84.7 percent resided in 1 to 4 member households. Almost three quarters of participants reported being educated beyond the high school level (74.4%). Over 62 percent had private dental insurance and 32 percent were uninsured. Almost 80 percent were in the upper middle and highest income brackets with only 5.5 percent being in the lowest income brackets (lower middle and lowest). Almost 85 percent perceived their oral health as good to excellent, and over 88 percent reported having oral pain rarely or never. Almost three quarters visited dental professionals more than once a year, while over 16 percent visited for emergency care only. Of those surveyed, 35.1 percent reported time loss from work, school or normal activities due to dental problems and treatment. Table 2 demonstrates that as income decreased so did the odds of reporting time loss, and as the frequency of experiencing oral pain increased, so did the likelihood of reporting time loss.

**Table 2:** Proportion and likelihood of reporting time loss

**Variable (N)**	**%**	**Unadjusted OR (95% CI)**	**P-value**
Age (yrs, N=29,141,400)			
6 to 11 (reference)	8.7		
12 to 19	14.6	1.2 (0.9, 1.6)	0.236
20 to 39	28.0	0.7 (0.5, 0.8)	0.001
40 to 59	33.3	0.8 (0.6, 1.0)	0.028
60 to 79	15.4	0.7 (0.5, 0.8)	0.001
Sex (N=29,141.400)			
Male (reference)	47.4		
Female	52.6	1.2 (0.9, 1.5)	0.123
Educational attainment (N=28,724,760)			
Greater than high school (reference)	74.3		
Less than high school	25.7	1.0 (0.8, 1.3)	0.940
Career status (N=24,986,201)			
Employed (reference)	53.7		
Student	20.0	1.4 (1.0, 2.0)	0.068
Unemployed	26.4	0.8 (0.6, 1.1)	0.093
Employment Type (N=20,091,451)			
Part-time (reference)	20.2		
Full-time	79.8	1.0 (0.8, 1.3)	0.756
Household size (N=29,141,400)			
1 to 2 people (reference)	42.7		
3 to 4 people	41.7	1.1 (0.9, 1.3)	0.298
5 or more people	15.6	1.1 (0.7, 1.6)	0.622
Aboriginal status (N=29,112,877)			
No (reference)	96.4		
Yes	3.6	1.3 (0.5, 3.5)	0.590
Immigrant status (N=29,139,278)			
No (reference)	80.3		
Yes	19.7	0.9 (0.6, 1.4)	0.565
Occupational classification (N=20,182,418)		1.0	
Management (reference)	9.4		
Business, finance and administrative	21.4	1.0 (0.6, 1.7)	0.835
Natural and applies sciences and related occupations	9.5	0.8 (0.4, 1.5)	0.438
Health occupations	5.1	0.6 (0.4, 0.9)	0.023
Occupations in social science, education, government service and religion	10.1	0.9 (0.5, 1.4)	0.491
Occupations in art, culture, recreation and sport	5.6	1.0 (0.6, 1.5)	0.923
Sales and service occupations	23.0	0.7 (0.4 ,1.1)	0.125
Trades, transport and equipment operators and related			
Occupations	11.1	0.5 (0.3, 1.0)	0.040
Occupations unique to primary industry	1.9	0.5 (0.2, 1.7)	0.251
Occupations unique to processing, manufacturing and utilities	2.9	0.4 (0.2, 0.9)	0.037
Insurance (N=28,964,287)			
Private insurance (reference)	71.2		
Public insurance	5.2	0.7 (0.5, 1.0)	0.045
No insurance	23.6	0.5 (0.4, 0.7)	0.000
Income adequacy (N=27,200,795)			
Highest income (reference)	55.9		
Upper middle income	29.8	0.7 (0.6, 0.8)	0.001
Middle income	10.6	0.5 (0.4, 0.6)	0.000
Lower middle income	2.8	0.5 (0.3, 0.7)	0.004
Lowest income	1.0	0.4 (0.2, 0.9)	0.037
Self-reported oral health (N=29,136,350)			
Good to excellent (reference)	85.9		
Poor to fair	14.1	0.8 (0.6, 1.2)	0.277
Self-reported oral pain (N=29,133,930)			
Rarely or never (reference)	84.1		
Sometimes	12.2	1.9 (1.4, 2.4)	0.001
Often	3.7	2.3 (1.1, 4.7)	0.026
Self-reported general health (N=29,137,886)			
Good to excellent (reference)	93.6		
Poor to fair	6.4	0.5 (0.4, 0.7)	0.001
Self-reported mental health (N=29,137,886)			
Good to excellent (reference)	93.6		
Poor to fair	6.4	0.5 (0.4, 0.7)	0.001
Self-reported disability status (N=28,856,199)			
No to mild disability (reference)	74.1		
Moderate to severe disability	26.0	1.1 (0.9, 1.2)	0.500
dmft prevalence (N=27,629,868)			
dmft=0 (reference)	94.5		
dmft>0	5.5	1.4 (1.1, 2.0)	0.021
DMFT prevalence (N=27,629,868)			
DMFT=0 (reference)	14.7		
DMFT>0	85.3	0.9 (0.7, 1.2)	0.584
Treatment needs (N=29,133,930)			
No (reference)	27.1		
Yes	72.9	1.5 (1.3, 1.9)	0.001
Preventive needs (N=29,133,930)			
No (reference)	90.1		
Yes	9.9	0.6 (0.5, 0.7)	0.001
Frequency of seeing dental professional (N=29,139,733)			
Less than once a year (reference)	3.5		
One or more times per year	92.3	5.4 (2.9, 9.9)	0.000
Emergency or never	4.2	0.6 (0.3, 1.2)	0.136
Time since last dental visit (N=28,196,229)			
Less than 1 year ago (reference)	97.1		
More than 1 year ago	2.9	0.04 (0.03, 0.06)	0.000

When looking at the impact that dental problems and treatment had on the sample, a mean of 3.5 hours per participant were lost from work, school, or normal activities due to dental problems and treatment, which equated to a total of 40.36 million hours at the population level. For adults (20–69 yrs), this equated to 4.14 million days lost, and for children (6–19 yrs), 2.27 million days lost. Table 3 shows that there were no significant differences between age groups in terms of the amount of time lost, while there was more than an hour difference between males and females (2.9 hrs vs. 4.2 hrs). Also, as the frequency of experiencing oral pain increased, so did the amount of time lost, with those experiencing frequent oral pain losing more than twice the amount of time than those who rarely or never experienced oral pain (7.5 hrs vs. 3.2 hrs). While not statistically significant, those who only sought professional care in emergency situations tended to lose more time than those who visited frequently (5.1 hrs vs. 3.5 hrs).

**Table 3:** Mean hours lost

**Variable**	**Mean hours lost (95% CI)**	**P-value**
Age (yrs)		
6 to 11	2.4 (2.1, 2.8)	
12 to 19	5.3 (3.3, 7.4)	
20 to 39	3.3 (2.7, 4.0)	
40 to 59	3.3 (2.5, 4.1)	
60 to 79	3.4 (2.9, 3.9)	0.407
Sex		
Male	2.9 (2.3, 3.4)	
Female	4.2 (3.5, 4.8)	0.015
Educational attainment		
Greater than high school	3.4 (3.0, 3.8)	
Less than high school	4.0 (2.8, 5.2)	0.352
Career status		
Employed	3.4 (2.8, 4.1)	
Student	5.2 (3.4, 7.1)	
Unemployed	3.0 (2.6, 3.5)	0.047
Employment type		
Part-time	4.3 (2.4, 6.1)	
Full-time	3.2 (2.7, 3.7)	0.286
Household size		
1 to 2 people	4.2 (3.6, 4.8)	
3 to 4 people	3.2 (2.8, 3.5)	
5 or more people	2.8 (2.0, 3.6)	0.004
Aboriginal status		
No	3.5 (3.2, 3.9)	
Yes	3.4 (1.7, 5.0)	0.833
Immigrant status		
No	3.6 (3.2, 4.0)	
Yes	3.4 (2.4, 4.4)	0.763
Occupational classification		
Management	2.9 (2.1, 3.7)	
Business, finance and administrative	3.8 (2.6, 5.1)	
Natural and applies sciences and related occupations	2.9 (2.1, 3.7)	
Health occupations	3.6 (1.1, 6.1)	
Occupations in social science, education, government service and		
Religion	3.7 (2.6, 4.8)	
Occupations in art, culture, recreation and sport	3.9 (1.9, 5.9)	
Sales and service occupations	3.7 (2.6, 4.9)	
Trades, transport and equipment operators and related		
Occupations	2.8 (2.0, 3.5)	
Occupations unique to primary industry	3.3 (2.4, 4.2)	
Occupations unique to processing, manufacturing and utilities	2.2 (1.7, 2.6)	0.450
Insurance		
Private insurance	3.6 (3.2, 4.1)	
Public insurance	2.8 (1.4, 4.1)	
No insurance	3.4 (2.8, 4.1)	0.498
Income adequacy		
Highest income	3.5 (2.9, 4.1)	
Upper middle income	3.9 (2.6, 5.1)	
Middle income	3.4 (2.9, 3.9)	
Lower middle income	3.9 (0.9, 7.0)	
Lowest income	2.7 (1.9, 3.5)	0.483
Self-reported oral health		
Good to excellent	3.4 (3.0, 3.7)	
Poor to fair	4.6 (3.2, 6.0)	0.105
Self-reported oral pain		
Rarely or never	3.2 (2.8, 3.6)	
Sometimes	4.9 (3.2, 6.6)	
Often	7.5 (4.7, 10.3)	0.022
Self-reported general health		
Good to excellent	3.6 (3.2, 3.9)	
Poor to fair	3.0 (2.3, 3.7)	0.114
Self-reported mental health		
Good to excellent	3.6 (3.2, 3.9)	
Poor to fair	3.0 (2.3, 3.7)	0.114
Self-reported disability status		
No to mild disability	3.4 (3.0, 3.8)	
Moderate to severe disability	4.0 (3.2, 4.8)	0.170
dmft prevalence		
dmft=0	3.6 (3.2, 3.9)	
dmft>0	2.8 (2.3, 3.4)	0.045
DMFT prevalence		
DMFT=0	4.0 (1.8, 6.2)	
DMFT>0	3.4 (3.0, 3.8)	0.614
Treatment needs		
Yes	3.6 (3.1, 4.1)	
No	3.4 (2.8, 4.1)	0.759
Preventive needs		
Yes	3.7 (2.6, 4.9)	
No	3.5 (3.1, 3.9)	0.753
Frequency of seeing dental professional		
Less than once a year	3.9 (2.2, 5.7)	
One or more times per year	3.5 (3.1, 3.8)	
Emergency or never	5.1 (2.0, 8.3)	0.448
Time since last dental visit		
Less than 1 year ago	3.6 (3.2, 3.9)	
More than 1 year ago	2.2 (1.1, 3.2)	0.019

Table 4 shows that those who reported experiencing oral pain often were almost 5.0 times more likely to report time lost compared to their counterparts, making oral pain the strongest predictor of reporting time loss. Table 5 shows that in terms of hours lost, being female was associated with a 1.3 hour increase in the amount of time lost when compared to males. While not statistically significant, being a student (at the university level) equated to 1.6 more hours lost when compared to employed individuals. Here again, as the frequency of experiencing oral pain increased so did the amount of time lost, with frequent oral pain being associated with an almost 4.0 hour increase in the amount of time lost.

**Table 4:** Multivariable logistic regression of what predicts reporting time loss (En Bloc model)

**Variables (N=18,153,248)**	**OR (95% CI)**	**P-value**
Age (yrs)		
6 to 19 (reference)		
20 to 39	0.9 (0.5, 1.6)	0.726
40 to 59	0.9 (0.4, 1.7)	0.666
60 to 79	1.3 (0.7, 2.2)	0.326
Sex		
Male (reference)		
Female	1.1 (0.8, 1.4)	0.638
Career Status		
Employed (reference)		
Student	1.3(0.7, 2.4)	0.402
Unemployed	0.8 (0.6, 1.2)	0.221
Occupational classification		
Management (reference)		
Business, finance and administrative	1.0 (0.6, 1.6)	0.932
Natural and applies sciences and related occupations	0.7 (0.4, 1.4)	0.261
Health occupations	0.5 (0.3, 0.9)	0.022
Occupations in social science, education, government service and		
Religion	0.6 (0.4, 1.0)	0.054
Occupations in art, culture, recreation and sport	0.6 (0.3, 1.4)	0.211
Sales and service occupations	0.6 (0.4, 0.9)	0.025
Trades, transport and equipment operators and related occupations	0.6 (0.3, 1.1)	0.098
Occupations unique to primary industry	1.0 (0.2, 3.9)	0.973
Occupation unique to processing, manufacturing and utilities	0.3 (0.1, 0.9)	0.04
Insurance		
Private insurance (reference)		
Public insurance	0.5 (0.1, 1.6)	0.205
No insurance	1.1 (0.7, 1.5)	0.758
Income adequacy		
Highest income (reference)		
Upper middle income	0.9 (0.7, 1.3)	0.704
Middle income	0.9 (0.5, 1.4)	0.531
Lower middle income	0.5 (0.2,1.2)	0.093
Lowest income	0.6 (0.1, 3.2)	0.055
Self-reported oral pain		
Rarely or never (reference)		
Sometimes	2.8 (1.6, 4.8)	0.001
Often	4.8 (2.2, 10.4)	0.001
Self-reported general health		
Good to excellent (reference)		
Poor to fair	0.5 (0.3, 0.8)	0.004
Treatment needs		
No (reference)		
Yes	1.1 (0.8, 1.5)	0.558
Preventive needs		
No (reference)		
Yes	1.4 (0.8, 2.5)	0.217
Frequency of seeing dental professional		
Less than once a year (reference)		
One or more times per year	1.2 (0.6, 2.4)	0.493
Emergency or never	1.2 (0.6, 2.7)	0.551
Time since last dental visit		
Less than 1 year ago (reference)		
More than 1 year ago	0.04 (0.02, 0.08)	0.001

**Table 5:** Multivariable linear regression of what predicts the amount of time lost (En Bloc model)

**Variables (N=9,489,782.7)**	**Observed coefficient (β, 95% CI)**	**P-value**
*Predisposing factors*		
Sex		
Male (reference)		
Female	1.28 (0.2, 2.4)	0.03
Career Status		
Employed (reference)		
Student	1.6 (−0.6, 3.8)	0.137
Unemployed	−0.5 (−1.2, 0.3)	0.211
*Needs factors*		
Self-reported oral health		
Good to excellent (reference)		
Poor to fair	0.76 (−0.6, 2.1)	0.235
Self-reported oral pain		
Rarely or never (reference)		
Sometimes	1.27 (−1.0, 3.5)	0.241
Often	3.88 (0.8, 7.0)	0.019
Self-reported general health		
Good to excellent (reference)		
Poor to fair	−0.45 (−1.4, 0.5)	0.322
*Health service use factors*		
Time since last dental visit		
Less than 1 year ago (reference)		
More than 1 year ago	−1.5 (−2.3, -0.3)	0.021
R^2^	0.0399	
F	F (8, 4) 2.60	
Prob>F	0.186	

Table 6 illustrates the potential productivity losses by occupation classification at both the individual and societal level. Individual losses are arguably minimal, ranging from almost $43 for those employed in processing, manufacturing and utilities, to over $110 for those employed in social science, education, government service and religion. When these losses are translated to the entire job sector, these losses become more substantial, with those employed in business, finance and administrative occupations, for example, having potential losses of over $230 million.

**Table 6:** Potential productivity losses due to dental problems and treatment at the individual and societal level

**Occupation classification**	**Mean hours lost**	**Potential individual losses ($)**	**Potential societal losses ($)**
Management	2.9	108.16	104,287,872
Business, finance and administrative	3.8	85.15	239,109,715
Natural and applies sciences and related occupations	2.9	95.17	103,278,484
Health occupations	3.6	97.44	97,790,784
Occupations in social science, education, government service and religion	3.7	112.51	165,333,445
Occupations in art, culture, recreation and sport	3.9	91.67	33,212,041
Sales and service occupations	3.7	58.72	220,857,664
Trades, transport and equipment operators and related occupations	2.8	64.51	131,064,967
Occupations unique to primary industry	3.3	76.39	16,439,128
Occupations unique to processing, manufacturing and utilities	2.2	42.96	32,232,888

## Discussion

This is the first study to use nationally representative data on reported time loss due to dental problems and treatment in the Canadian population. These findings are integral to understanding the impact of dental problems and treatment at the societal level, and to the inclusion of oral health in broader health policy debates, especially because of a renewed interest in the economic implications of illness [19]. This study found that 39 percent of participants, representing over 13 million Canadians, reported time loss from work, school or normal activities due to dental problems and treatment. Among participants who reported time loss, the majority were from middle- to high-income groups. This is consistent with the findings of Reisine and Miller (1985), who reported that in a sample of Americans, those with greater financial resources were more likely to miss work for dental visits [20]. Also, given the structure of American and Canadian oral health care systems (i.e. almost wholly financed and delivered privately on a fee for service basis), this finding is not surprising considering that utilizing and accessing dental care is largely determined by an individual’s ability to pay [21]. In this regard, the current study found that 71 percent of those with private insurance reported time loss compared to only 23 percent of those without dental insurance. When amount of time loss was quantified, the mean number of hours lost per participant was arguably inconsequential at 3.5 hours, however the total number of hours lost at the societal level was estimated at over 40 million, arguably representing a substantial impact. This study also found that experiencing oral pain often was associated with a 4-hour increase in time lost, which is consistent with the literature, which states that pain is often associated with reporting time loss and losing more time as the treatments required are more extensive [6]. Oral pain is the strongest predictor of reporting time loss and is associated with more lost time. As Ramraj (2012) reported those within the lowest income brackets and those without insurance were over 2 times more likely to have surgical treatment needs, which is often preceded by pain [22]. This is consistent with the finding of Quiñonez et al. (2010) who reported that those in the lowest income brackets, without dental insurance, that experienced oral pain, and that had visited a hospital emergency department in the past due to a dental problem, were all more likely to report a disability day due to a dental problem [23].

This study’s finding also highlights the importance of “good” and “bad” time loss (e.g., time lost for check-ups and preventive care vs. for major restorative or surgical care). This concept becomes important in the realm of policy and insurance decisions. For example, from an employer’s viewpoint, investments in prevention and accessible care for all employees would likely mitigate both “bad” time loss and potential productivity losses due to dental problems and treatment. In terms of insurance decisions, amending coverage to include prevention may reduce overall costs by reducing the need for more complex and costly treatments. This brings to the surface the idea that by raising the financial eligibility level for current public programs, this would also allow those segments of the population not currently covered increased access to care (e.g., the working poor), further mitigating “bad” time loss. At the policy level this is consistent with prevention strategies for other conditions (i.e., back injuries where protocols are used to prevent or minimize the risk of injury at work), and provides another opportunity for dentistry to be discussed within broader health policy.

As mentioned in the introduction, despite discussion surrounding the indirect costs of medical conditions, there are very few examples of this in the dental literature [3,6,20,23]. These discussions have focused on the differences in time loss (i.e., days lost from work or disability days) by occupational class (e.g., blue versus white collar jobs) and by job autonomy, such that those with white collar jobs or greater autonomy tended to lose more time, albeit for different reasons than those with blue collar jobs or less autonomy [3,6]. The availability of labour force data in the CHMS, which corresponded to the wage data of the Canadian Labour Force Survey (LFS) allowed for further examination of this relationship and to monetize these potential losses. This study found that the potential productivity losses attributed to time lost from work were over $1 billion. This is likely an underestimation of the overall productivity losses as non-market losses were not valued (time from school or normal activities). Yet by quantifying these losses, this study arguably provides a starting point for discussions on the economic importance of oral health, while providing policymakers with a better understanding of the true cost of dental problems and treatment in the Canadian population.

Comparison between productivity losses due to dental problems and treatment and other illnesses were undertaken, in an attempt to bridge a key knowledge gap in the understanding of the burden of dental problems and treatment and its magnitude compared to other illnesses. The potential losses attributed to dental problems and treatment ($1,143,606,988) were found to be most comparable to those resulting from musculoskeletal strains and sprains ($1,250,947,561), and bone disorders ($1,482,048,535) [24]. Nonetheless, strong caveats accompany this comparison. First, a complete economic analysis was not undertaken here and only similar costs (i.e., lost wages and morbidity) were compared.

Direct comparison to the United States is difficult due to the vastly different recall periods of the U.S. National Health Interview Study (1996, NHIS) and the CHMS (2 weeks vs. 12 months). The NHIS survey queried time loss for personal dental care, accompanying a family member for dental care and additional impacts on normal activities. Comparatively the CHMS merely asked if time was lost from any aspect of daily living in the previous 12 months. More comparable are the findings of the recent Australian National Dental Telephone Interview Survey (2010, NDTIS), which asked participants to report time loss from work/school and restricted activity separately, in the previous 12 months due to dental problems. The study found that those who reported their oral health as poor to fair reported losing more time than those with good to excellent oral health [5]. The authors stated that for a segment of the population the cycle of poor oral health and problem oriented visiting warranted further attention [5]. The authors also estimated $836.5 million (CDN) in losses due to missed work and restricted activity due to oral health problems, a finding similar to the losses estimated in this study [5]. Ultimately, policy changes aimed to relieve pressures faced by those burdened with the most time loss (the poor), such as raising the financial threshold for public programs, improving the quality of insurance offered to more junior employees, or subsidizing dental treatment in some form, may also reduce the impact of dental problems and treatment on society through concomitant productivity losses.

It is important to understand the limitations of this study. Most significant is the limited way in which the question regarding time loss was structured. For example, this study was unable to discern which aspect of daily living was impacted (work, school, or normal activities) and what the underlying reasons for seeking care were; both of which are required to better understand the dynamics and quality of time lost in the population. This inability to discriminate between time loss for treatment, which could mostly be due to preventive reasons, and that due to pain or problems may explain why those in higher income brackets reported more time loss. This may also explain why individuals with a DMFT=0 lost more time than those with a DMFT>0 as seen in Table 3. Thus, it is possible that the majority of time loss reported in this study was due to check-ups or preventive services and that those with significant dental problems did not report losing as much time due to dental issues, perhaps due to barriers to care. This reasoning is consistent with what is known about social gradients in oral health, the use of dental care, and access to dental care, in North America. In this light, it was assumed that for every adult reporting time loss, the loss was from work and that every school-age individual reporting lost time did so from school.

The overall participation in the CHMS was low (51.7%) and as such multiple biases are likely. First, with the voluntary nature of the CHMS, those who participated may be inherently different from those who chose not to participate, thus reducing the generalizability of the data. Second, the combined length of the household questionnaire (722 questions) and the subsequent clinic visit may have acted as a deterrent for participation. Further to this, for those who participated, social desirability bias may have also played a role. Here, participants could have responded with answers they believed could show them in a good light, or allowed them to complete the questionnaire more quickly.

For the calculation of potential productivity losses (at the societal level) it was assumed that each and all job sector employees visited a dental professional in the last 12 months. Also a complete economic analysis was not completed, thus the estimate of productivity losses reflects only those that were assumed to be attributable to work loss (i.e. time lost for employed persons only). In light of these limitations it is suggested that future cycles of the CHMS include separate questions for time loss from work, school or normal activities. Also, questions that clarify the underlying reasons for seeking professional care would allow for better policy information. For example, collection of data regarding time loss from medical and/or other issues (as the NHIS does) may yield common reasons for time loss (e.g., pain), may strengthen an understanding of the relationship between oral health and general health, and may identify factors which are amenable to policy change. It may be that investments outside of the health care sector yield the largest returns for improving both general and oral health (e.g., increased access to quality employment or child care), thereby eliminating competing economic stressors and increasing the uptake of dental services (more discretionary income and time, which has been associated with increased utilization of dental services, for example). Finally, a complete economic analysis of the impacts of dental problems and treatment on Canadian society would allow for ease of comparison to medical conditions and would remove some of the caveats associated with this study’s estimate of potential productivity losses. A complete economic analysis would also provide more detailed information regarding the total burden of dental problems and treatment and could be used as justification for increased funding or more efficient use of dental care dollars by directing investments towards programs and infrastructure that would mitigate these losses.

## Conclusions

This study has shown that, potentially, over 40 million hours are lost annually due to dental problems and treatment in Canada, with subsequent potential productivity losses of over $1 billion dollars. These losses are comparable to those experienced for other illnesses (e.g., musculoskeletal sprains). Further investigation into the underlying reasons for time loss, and which aspects of daily living are impacted by this time loss, are necessary for a fuller understanding of the policy implications associated with the economic impacts of dental problems and treatment in Canadian society.

## Abbreviations

CHMS: Canadian Health Measures Survey; OECD: Organization for Economic Cooperation and Development; ARCPOH: Australian Research Centre for Population Oral Heath; CV: Coefficient of Variance; RDC: Research Data Centre; VIF: Variation Inflation Factor; LFS: Labour Force Survey; NHIS: National Health Interview Study; NDTIS: National Dental Telephone Survey.

## Competing interests

The authors declare they have no competing interests.

## Authors’ contributions

AH, CQ, LD, and AA contributed to the study conception and design. AH was granted access to the RDC for data analysis and interpretation. VR provided assistance with the analysis and interpretation. CQ revised the manuscript critically for intellectual content. All authors reviewed, read and approved the final manuscript.

## Pre-publication history

The pre-publication history for this paper can be accessed here:

http://www.biomedcentral.com/1472-6831/13/17/prepub
